# Efficacy of clinical evaluations for COVID-19 on the front line

**DOI:** 10.1186/s12245-020-00313-w

**Published:** 2020-11-07

**Authors:** Lili L. Barsky, Joseph E. Ebinger, Mona Alotaibi, Mohit Jain, Sam Torbati, Bradley T. Rosen, Susan Cheng

**Affiliations:** 1 Department of Cardiology, Smidt Heart Institute, Cedars-Sinai Medical Center, 127 S. San Vicente Boulevard, Los Angeles, CA 90048 USA; 2 Barbra Streisand Women’s Heart Center, Cedars-Sinai Medical Center, 127 S. San Vicente Boulevard, Los Angeles, CA 90048 USA; 3 Department of Pulmonology, University of California San Diego, 200 W. Arbor Drive, San Diego, CA 92103 USA; 4 Department of Medicine and Pharmacology, University of California San Diego, 200 W. Arbor Drive, San Diego, CA 92103 USA; 5 Department of Emergency Medicine, Cedars-Sinai Medical Center, 8700 Beverly Boulevard, Los Angeles, CA 90048 USA; 6 Inpatient Specialty Program, Cedars-Sinai Medical Center, 8700 Beverly Boulevard, Los Angeles, CA 90048 USA

**Keywords:** Clinical assessment, Effectiveness, COVID-19

To the Editor:

In the midst of the COVID-19 pandemic, there remains limited availability of Food and Drug Administration-approved tests for presence of the SARS-CoV-2 agent [1, 2]. Even as testing capacity expands, optimization of resource utilization in the healthcare setting remains a significant priority [3, 4]. Thus, the vast majority of front line work being done to evaluate for possible COVID-19 is highly dependent on the clinical assessment of a presenting patient’s signs and symptoms. The extent to which current clinical assessments are effective, in the era of rapidly evolving local and professional guidelines, is not entirely clear.

We conducted a retrospective review of patients assessed for possible COVID-19 illness at our urban medical center in Los Angeles, California. The institutional review board deemed the study exempt. We carefully reviewed all clinical records to ascertain the provider’s level of clinical suspicion for COVID-19 illness and compared these assessments with available results of SARS-CoV-2 testing, in addition to longitudinal data on clinical outcomes. We found that the vast majority of patients (96% of *N* = 25) clinically assessed to have a low probability of COVID-19 illness were subsequently confirmed to have either a negative SARS-CoV-2 test result or, in the absence of testing, clinical stability without any further concern for COVID-19 illness (Table 1). All clinical assessments were performed by a physician, with some (16%) conducted by a nurse practitioner or physician assistant in conjunction with physician supervision.

**Table 1:** Characteristics, testing status, and clinical outcomes for *N* = 25 patients

Age	Sex	Major comorbidities	Presenting symptoms	Vital signs	Lab/imaging findings	Most likely differential/explanation	COVID-19 index of suspicion	COVID-19 test status	Outcome(s)
40	F	Malignancy with pulmonary metastasis	SOB, cough	Afebrile Baseline oxygen saturation	CT with progression of pulmonary metastasis	Progression of pulmonary metastasis	LOW	NOT DONE	***Deceased***—unclear whether due to metastasis or undiagnosed COVID-19
80	M	Malignancy with pulmonary metastasis	SOB, cough	Afebrile Baseline oxygen saturation	Elevated D dimer CXR with unilateral infiltrates CT with pulmonary embolism	PE with possible post-obstructive pneumonia	LOW	NEG	Anticoagulated Given antibiotics Discharged home
77	F	End stage renal disease Heart failure	SOB, cough	Afebrile Baseline oxygen saturation	CXR with bilateral infiltrates ***BUT*** Elevated BNP	Heart failure exacerbation	LOW	NOT DONE	Diuresed Discharged home
65	F	Breast cancer Nephrostomy tubes Chronic pleural effusion	Fevers, SOB, flank pain	Hypotension Afebrile Normal oxygen saturation	UTI CXR unchanged	Urosepsis	LOW	NOT DONE	Given antibiotics Nephrostomy tubes replaced Discharged home
63	F	Hyponatremia	Subjective fevers, weakness	Afebrile Normal oxygen saturation	Acute on chronic hyponatremia	Acute on chronic hyponatremia due to increased dose of thiazide	LOW	NOT DONE	Intravenous fluids given Thiazide discontinued Discharged home
105	M	Large pulmonary nodules	SOB, cough, coryza	Afebrile Baseline oxygen saturation	CXR and CT unchanged	Respiratory bronchiolitis	LOW	NOT DONE	Given PRN antitussives and nebulizer treatments Discharged home
77	F	Renal transplant Diabetes mellitus Coronary artery disease	Encephalopathy	Hypotension Afebrile Normal oxygen saturation	Flu positive CXR and CT unchanged	Flu vs. BK viremia	LOW	NEG	Given Tamiflu Discharged home ***Delay in neurologic evaluation (lumbar puncture, EEG) while PUI***
86	F	Diabetes mellitus Chronic kidney disease	SOB, cough	Afebrile Normal oxygen saturation	CXR with unilateral infiltrates Elevated procalcitonin	Community-acquired pneumonia	LOW	NOT DONE	Given antibiotics Discharged home
45	M	Pancreatic cancer	Cough	Febrile Hypotensive Hypoxic	CXR with unilateral infiltrates D dimer elevated Lactic acidosis	Community-acquired pneumonia	LOW	NEG	Given antibiotics Discharged home
65	F	Lupus Active recurrent pericarditis	SOB, weakness	Afebrile (though on steroids for pericarditis) Normal oxygen saturation	Leukocytosis ***BUT*** CXR unchanged	Sequela of active pericarditis vs. steroid use	LOW	NEG	Given empiric antibiotics Discharged home ***TTE deferred while PUI***
71	F	Chronic angina Chronic SOB Myocardial infarction Hypertension	Acute on chronic angina, SOB, pharyngitis	Afebrile Normal oxygen saturation	CXR unchanged Troponemia ECG unchanged	Acute coronary syndrome vs. anxiety	LOW	NEG	Discharged home ***CCTA was done, but this was deferred while PUI***
46	F	Heart failure Anxiety	Acute on chronic SOB	Afebrile Normal oxygen saturation	CXR unchanged Elevated BNP	Heart failure exacerbation	LOW	NEG	Diuresed Discharged home
60	F	Diverticulitis Partial bowel resection with ostomy	Abdominal pain, nausea, vomiting	Afebrile Normal oxygen saturation	CXR unchanged Severe acute kidney injury Severe hyperkalemia	Metabolic derangement due to delayed ostomy revision	LOW	NEG	Hyperkalemia treated Given fluids Ostomy bag revised Discharged home ***Ostomy bag revision had been delayed while PUI***
75	F	Irritable bowel syndrome – diarrhea type	Nausea, acute on chronic diarrhea	Afebrile Normal oxygen saturation	CXR unchanged Hypokalemia	Viral enteritis	LOW	NEG	Given fluids Potassium repleted Discharged home
83	M	Atrial fibrillation Heart failure Chronic sinusitis	SOB, abdominal pain, diarrhea, fall	Afebrile Normal oxygen saturation	Lymphopenia ***BUT*** CT with stercoral colitis Acute hyponatremia CXR unchanged	Stercoral colitis	LOW	NEG	Given fluids Discharged home ***PT/OT needed for fall deferred while PUI***
49	M	Bronchiectasis Multiple myeloma	Fever, cough, SOB	Febrile Baseline oxygen saturation	Lymphopenia ***BUT*** CXR unchanged	Community-acquired pneumonia	MOD	NEG	Improved on antibiotics Discharged home
66	M	End stage renal disease on peritoneal dialysis Coronary artery disease Diabetes mellitus	Abdominal pain nausea, vomiting	Afebrile Baseline oxygen saturation	CT with ground glass opacities concerning for pulmonary edema Normal BNP	Peritonitis	LOW	NEG	Given antibiotics Discharged home ***Testing ordered on basis of CT results, not symptoms***
53	F	Heart transplant	SOB, abdominal pain	Afebrile Baseline oxygen saturation	CXR with bilateral infiltrates ***BUT*** Elevated BNP	Heart failure	LOW	NEG	Diuresed ***Admission to transplant service delayed while PUI***
90	F	Chronic bilateral pleural effusions Failure to thrive	SOB, weakness, dysuria	Afebrile Baseline oxygen saturation	CXR unchanged UTI	UTI Deconditioning	LOW	NEG	Given antibiotics ***PT/OT needed for deconditioning deferred while PUI***
84	F	Shoulder dislocation Mitral regurgitation	Shoulder pain	Afebrile Normal oxygen saturation	XRAY with shoulder dislocation	Shoulder dislocation	LOW	NEG	***Orthopedic surgery delayed while PUI***
78	M	Hypertension	Cough, melena	Afebrile Normal oxygen saturation	Acute blood loss anemia CXR unchanged	Acute blood loss anemia	LOW	NEG	Given blood transfusion ***Esophagogastroduodenoscopy delayed while PUI***
72	M	Invasive gastric cancer Diabetes mellitus	Chest pain	Afebrile Baseline oxygen saturation	CXR unchanged No troponemia No ECG changes	Deconditioning	LOW	NEG	Admitted to discuss treatment for gastric cancer ***Treatment delayed while PUI***
30	F	Portal vein thrombosis	Nausea, abdominal pain	Afebrile Normal oxygen saturation	Elevated liver enzymes US and CT with gallbladder sludge	Cholecystitis	LOW	NEG	Given fluids ***Cholecystectomy deferred while PUI***
81	F	Dementia with psychosis Recurrent UTI Diabetes mellitus Aspiration pneumonia	Altered mental status, displaced G-tube	Afebrile Normal oxygen saturation	UTI CXR unchanged	UTI Displaced G-tube	LOW	NEG	Given fluids and antibiotics G-tube replaced
54	F	Poorly controlled hypertension	Angina	Severe hypertension Afebrile Normal oxygen saturation	Troponemia CXR unchanged	Hypertensive emergency with NSTEMI	LOW	NEG	Controlled blood pressure ***Catheterization delayed while PUI***

*SOB* shortness of breath, *NEG* negative, *CXR* chest xray, *UTI* urinary tract infection, *CT* computed tomography, *PRN* as needed, *PE* pulmonary embolism, *PPE* personal protective equipment, *PUI* person under investigation, *TTE* transthoracic echocardiogram, *PT/OT* physical therapy/occupational therapy, *US* ultrasound, *NSTEMI* non-ST elevation myocardial infarction

In the absence of widespread readily available access to SARS-CoV-2 testing, clinical assessment is and will remain the standard of care for initially determining probability of COVID-19 illness and, in turn, appropriateness for receiving testing—especially in areas where testing availability is limited. This case series from an urban medical center suggests that despite the rapidly evolving body of knowledge around COVID-19 illness and its variable presentations among affected patients, clinical provider assessment of high versus low probability of active infection can be relatively reliable. This case series further supports the hypothesis that a well-informed clinical assessment, with or without concurrent access to rapid point-of-care SARS-CoV-2 testing, could be leveraged to more efficiently triage patients [5]—even those with medical comorbidities whose chronic illness burden may appear to pose a diagnostic challenge at the outset. In effect, a clinical evaluation that does not rely on viral testing results may be very accurate and substantially aid in ongoing efforts to conserve and appropriately prioritize the use of medical resources. Use of sound clinical judgment can also facilitate consideration of alternative diagnostic explanations.

## Data Availability

All data generated or analyzed during this study are included in this published article.
